# The Emergence of Model Systems to Investigate the Link Between Traumatic Brain Injury and Alzheimer’s Disease

**DOI:** 10.3389/fnagi.2021.813544

**Published:** 2022-02-08

**Authors:** Gayathri Srinivasan, David A. Brafman

**Affiliations:** School of Biological and Health Systems Engineering, Arizona State University, Tempe, AZ, United States

**Keywords:** traumatic brain injury, Alzheimer’s disease, *in vivo* models, *in vitro* models, pluripotent stem cells, genome engineering

## Abstract

Numerous epidemiological studies have demonstrated that individuals who have sustained a traumatic brain injury (TBI) have an elevated risk for developing Alzheimer’s disease and Alzheimer’s-related dementias (AD/ADRD). Despite these connections, the underlying mechanisms by which TBI induces AD-related pathology, neuronal dysfunction, and cognitive decline have yet to be elucidated. In this review, we will discuss the various *in vivo* and *in vitro* models that are being employed to provide more definite mechanistic relationships between TBI-induced mechanical injury and AD-related phenotypes. In particular, we will highlight the strengths and weaknesses of each of these model systems as it relates to advancing the understanding of the mechanisms that lead to TBI-induced AD onset and progression as well as providing platforms to evaluate potential therapies. Finally, we will discuss how emerging methods including the use of human induced pluripotent stem cell (hiPSC)-derived cultures and genome engineering technologies can be employed to generate better models of TBI-induced AD.

## Introduction

Alzheimer’s disease (AD) affects over 6 million individuals in the U.S. and has a direct cost estimated in excess of $355 billion/year (Alzheimer’s Association, 2021). Although a vast majority of late onset AD (LOAD) cases are sporadic, numerous genetic and environmental risk factors have been identified that contribute to lifetime risk of developing the disease. In this vein, studies over the past few decades have also implicated traumatic brain injury (TBI) in the onset and progression of neurodegenerative diseases later in life (Schofield et al., 1997; Chen et al., 2007; Gardner et al., 2018). Indeed, it has been generally accepted that in addition to the primary mechanical damage inflicted, TBI sets in place various secondary injury mechanisms with acute and long-term effects. In addition, several studies have shown that a large-percentage of TBI patients not only suffer from short-term cognitive impairment but also experience long-term cognitive decline similar to that observed in AD patients (Rabinowitz and Levin, 2014). Furthermore, pathologies associated with AD have been observed in post-mortem brain tissue of TBI patients (Ikonomovic et al., 2004).

Despite these associations between a TBI and AD onset, the mechanisms by which a TBI induces or augments AD-related neurodegenerative processes are unclear. In this review, we will first discuss the various epidemiological and clinical evidence that exists for TBI-induced AD. Next, we will describe the various potential mechanisms for TBI-induced AD that have been suggested by these studies. Finally, we will discuss the emergence of *in vitro* models of TBI and speculate about how these can be used to further elucidate the mechanisms by which TBI induces AD-related pathology, neuronal dysfunction, and cognitive decline.

## Evidence for Traumatic Brain Injury-Induced Alzheimer’s Disease

Because cognitive impairment often follows a TBI, the onset of AD following head trauma has been an active area of investigation. In this section, we will review the recent epidemiological, clinical, and animal-based studies that have investigated this relationship between TBI and AD. In addition, we will discuss the limitations of these studies as it relates to providing a definitive mechanistic link between TBI and age-related progression of AD.

### Epidemiological Studies

Broadly speaking, epidemiological studies investigating the link between TBI and AD have been divided in their conclusions with some demonstrating an increased risk of AD post-TBI while others have not identified a significant association (Lye and Shores, 2000; Dams-O’Connor et al., 2016). With respect to the studies that support the link between TBI and AD, it has been reported that TBI approximately doubles the likelihood of an individual developing AD (Fleminger et al., 2003). In support of these studies, it has been suggested that after age and Apolipoprotein (E) genotype, TBI is the strongest risk factor associated with non-familial, sporadic forms of AD (Daviglus et al., 2011; Armstrong, 2019). Likewise, TBI has also been reported to accelerate the age of onset of AD (Nemetz et al., 1999; Schaffert et al., 2018). However, it should be noted that additional studies have suggested that several factors might modulate this risk of TBI-induced AD. For example, the severity of injury has a significant effect on the likelihood of development of AD with the reported risk increasing only twofold with moderate TBI but over fourfold with severe TBI (Guo et al., 2000; Plassman et al., 2000). Likewise, some studies report an increased AD risk only in TBI cases with loss of consciousness (Schofield et al., 1997; Guo et al., 2000).

It also has been suggested that the risk of AD after a TBI might be sex-dependent with a significant association only found in men (Fleminger et al., 2003; Weiner et al., 2017). This is interesting given that women have a higher risk of developing AD in the absence of head injury (Viña and Lloret, 2010; Laws et al., 2018; Guo et al., 2022). It has been suggested that female hormones such as estrogen and progesterone exert protective effects post-TBI by modulating anti-inflammatory and antioxidant processes (Vagnerova et al., 2008; Brotfain et al., 2016; Ma et al., 2019). In the same vein, these same sex-related hormones may alter AD-related mechanisms post-injury as well. However, others have suggested that sex-based differences in clinical outcomes after TBI might be independent of biological-related mechanisms but rather due to sex differences in injury type or treatment post-TBI (Ma et al., 2019; reviewed here Mollayeva et al., 2018). Moving forward, establishing definitive correlations between sex and TBI-induced AD will require more extensive, well-controlled studies (Ma et al., 2019).

Finally, as is the case with many epidemiological studies, there are several limitations that should be noted. For example, many of the studies cited are retrospective in nature, introducing recall bias due to which the association between TBI and AD might be significant (Weiner et al., 2017). Moreover, many of these studies suffer from the well-documented limitations of diagnosing AD using only neurophysiological profiling or cognitive examination (Tarawneh and Holtzman, 2012; Dubois et al., 2016; Weiner et al., 2017). Perhaps because of these limitations, some prospective studies have failed to find significant association between TBI and AD (Katzman et al., 1989; Launer et al., 1999; Mehta et al., 1999; Lindsay et al., 2002) even in cases with loss of consciousness (Crane et al., 2016).

### Clinical Studies

Clinicopathological studies have demonstrated a link between TBI and the development of AD-related pathologies such as the formation of amyloid beta (Aβ) plaques and neurofibrillary tangles (NFTs) consisting of hyperphosphorylated tau (p-tau). While amyloid precursor protein (APP) accumulation is a well-known marker for diffuse axonal injury (DAI) (Gentleman et al., 1993), a major pathology of TBI, evidence for Aβ accumulation post-injury first appeared in studies by Clinton et al. (1991) and Roberts et al. (1991) where Aβ deposits were observed in one-third of short term survivors of TBI within days of the injury. Moreover, these deposits were observed up to a few years after the injury in one-third of TBI survivors (Roberts et al., 1994). Similarly, Aβ42 containing diffuse Aβ deposits have been observed acutely after injury (Gentleman et al., 1997). While Aβ plaques similar to those observed in AD patients have been reported in a few long-term survivors of TBI (Johnson et al., 2012), some studies report a lack of dense mature plaques in short-term (Ikonomovic et al., 2004) as well as long-term survivors of TBI (Chen et al., 2009). It has been suggested that clearance of Aβ deposits post injury by the enzyme neprilysin might explain this lack of Aβ plaques in long-term TBI survivors (Chen et al., 2009). Similarly, although the enzymes involved in the amyloidogenic processing of APP such PS1 and BACE1 were found to colocalize with Aβ in damaged axons, plaque formation was not observed even years after the injury (Uryu et al., 2007). Overall, although the evidence for Aβ plaques post-injury is unclear, these studies demonstrate increased amyloid burden in TBI patients.

Repetitive mild TBI in in athletes (Stern et al., 2019), boxers (McCrory et al., 2007) and veterans (McKee et al., 2013) has been identified as a risk factor for the development of a tauopathy, known as chronic traumatic encephalopathy (CTE). On the other hand, the effect of a single TBI on tau pathology is yet to be understood (Collins-Praino and Corrigan, 2017). In fact, NFTs have been only rarely, if at all, reported in acute survivors of TBI (Smith et al., 2003; Ikonomovic et al., 2004) although alterations in tau immunoreactivity even in the absence of NFTs have been observed and warrant further studies. For instance, after injury, accumulation of cis-p tau, the conformation of p-tau prone to aggregation and found in NFTs in AD patients, has been reported (Albayram et al., 2017). In contrast to studies focused on acute effects of injury, NFTs, tau deposition and white matter degeneration have been reported in long-term survivors of TBI as indicated by immunohistochemical studies (Johnson et al., 2012, 2013). Tau deposits in long-term survivors of TBI have also been correlated with onset of neuropsychiatric conditions later in life (Takahata et al., 2019).

Overall, while clinical studies have provided important evidence for the connection between TBI and subsequent AD onset, the majority of these studies are focused on the examination of post-mortem tissue which only provide an endpoint of the disease. As such, it is difficult to establish temporal relationships between the initial TBI and formation of AD-related pathologies. Recently, progress has been made in the use of biomarkers and advanced imaging techniques to determine such connections. For example, amyloid PET imaging in long-term survivors of TBI revealed amyloid pathology in patients after brain trauma albeit in a pattern different from those observed in AD patients (Scott et al., 2016). As it relates to tauopathy, PET scans have revealed tau deposition in long-term survivors of TBI (Gorgoraptis et al., 2019). Along similar lines, cleaved tau in cerebrospinal fluid (CSF) and total tau in interstitial fluid (ISF) have been observed to increase post-injury and have been suggested as biomarkers for TBI (Gabbita et al., 2005; Marklund et al., 2009).

### Animal Models of Injury

Because of the limitations of epidemiological and clinical studies, animal models have been employed to identify potential mechanistic links between TBI and the development of AD. Broadly speaking, these studies have employed various AD transgenic mouse models (e.g., 3xTg, APP/PS1, Tg2576, PS19, APP knockin) in the context of numerous injury systems [e.g., controlled cortical impact (CCI), midline fluid percussion injury (mFPI), lateral fluid percussion injury (LFPI), closed head impact model of engineered rotational acceleration (CHIMERA)]. These studies are too numerous to review here but have been summarized in Table 1. Instead, we highlight some of the key studies that demonstrate a link between TBI and AD.

**TABLE 1:** Summary of AD phenotypes observed in animal models of injury.

Animal model	Type of transgenic animal	TBI model	TBI induced at	Time post-injury	Key conclusions	References
Mouse	3x-Tg	CCI	5–7 months	24 h, 7 days	↑ Aβ and p-tau	Tran et al., 2011a
	Tau P301L	CCI	6 months	24 h	↑p-tau	Tran et al., 2011b
	APP/PS1	Weight drop	12–16 months	72 h	↑Neuronal loss ↑microglial reactivity	Lecca et al., 2019
	APP/PS1	CHIMERA	6 or 13 months	14 days	Transient ↑Aβ deposit in young mice, ↓Aβ deposit in old mice ↑microglial reactivity in old WT mice ↓ in APP/PS1 mice	Cheng et al., 2018
	APP/PS1	CHIMERA	5.7 months	8 months	↑Microglial and astrocytic reactivity No changes in Aβ or tau levels	Cheng et al., 2019
	APP/PS1	Focal (needle) injury	3 or 9 months	24 h, 7 days	No changes in Aβ plaque due to injury ↑Microglial reactivity and ↓Synaptophysin at 24 h, recovered by 7 days in both WT and APP/PS1 mice	Collins et al., 2015
	APP/PS1	CCI	3 months	16 weeks	↓Aβ deposition	Miszczuk et al., 2016
	APP/PS1	CCI	3 months	2, 6 weeks	↑Aβ deposits ↑neuronal loss	Tajiri et al., 2013
	APP/PS1	CCI	8 months	9 h-2 months	↑Inflammation at 2 months post-injury	Webster et al., 2015
	hTau	FPI	2 months	3 days, 135 days	↓Microglial reactivity	Kokiko-Cochran et al., 2017
	PDAPP	CCI	2 year	1, 9, 16 weeks	↓In Aβ deposits ↑neuronal loss at 16 weeks	Nakagawa et al., 2000
	PDAPP	CCI	3–4 months	2 weeks	↑Cognitive dysfunction	Brody and Holtzman, 2006
	PDAPP	CCI	4 months	2, 5, 8 months	↓In Aβ deposits ↑neuronal loss at 5–8 weeks	Nakagawa et al., 1999
	TgArcSwe	CCI, mFPI	3 months	12, 24 weeks	↑Aβ deposits ↑ reactive astrocytes	Zyśk et al., 2019
	R1.40	LFPI	2 months	3, 120 days	↓Inflammation at 3 dpi ↑ at 120 dpi	Kokiko-Cochran et al., 2015
	Non-transgenic (BALB/c)	mFPI	3 months	7, 30 days	LPS challenge 30 dpi ↑inflammation and cognitive deficits	Muccigrosso et al., 2016
	Non-transgenic (C57BL/6)	CCI		3 months	↑Microglial mGluR5 expression which when inhibited ↓neuronal loss	Byrnes et al., 2012
	Non-transgenic (C57BL/6)	Weight drop	3 months	4 h–30 days	↑γ-Secretase expression in astrocytes and microglia	Nadler et al., 2008
Rat	Non-transgenic (Sprague-Dawley)	CCI	2 months	3 h–4 weeks	↑APP	Ciallella et al., 2002
		FPI		1–30 days	Transient/delayed ↑APP	Bramlett et al., 1997
		Compression		1–21 days	↓APP in injury periphery ↑ in white matter	Lewén et al., 1995
Pig		Rotational acceleration	4 months	1–10 days	↑Diffuse Aβ deposits ↑tau	Smith et al., 1999a

*CCI, Controlled Cortical Impact; FPI, Fluid Percussion Injury; mFPI, Midline Fluid Percussion Injury; LFPI, Lateral Fluid Percussion Injury; CHIMERA, Closed-Head Impact Model of Engineered Rotational Acceleration; 3x-Tg, Triple-Transgenic; APP, Amyloid Precursor Protein; PS1, Presenilin-1; hTau, Humanized Tau; PDAPP, PDGF-Driven Human APP; TgArcSwe, Transgenic with Arctic mutation and Swedish mutation.*

With respect to amyloid-related phenotypes, increased APP immunoreactivity, a marker of axonal damage has been observed in the white matter in rats acutely (Ciallella et al., 2002) as well as chronically (Pierce et al., 1998; Acosta et al., 2017) (up to 1 year) post-injury. It is important to note that the temporal and spatial pattern of APP immunoreactivity in the gray matter, however, seem to vary depending on the mode of injury as well as its severity (Bramlett et al., 1997). For instance, in a rat CCI model, increased APP immunoreactivity was observed in gray matter 6 months post-injury (Acosta et al., 2017) whereas in an FPI model, the immunoreactivity increased transiently in the striatum but remained elevated at the site of injury up to 1 month (Bramlett et al., 1997). Similarly, in a rat mFPI model APP immunoreactivity was observed to increase acutely in the hippocampus but decrease by 7 days post-injury which could be attributed to the cell death observed at this time point (Murakami et al., 1998). Several transgenic mouse models of AD that express human APP, such as 3x-Tg and APP/PS1 have shown acute intra-axonal Aβ accumulation (Tran et al., 2011b). APP/PS1 mice have also shown to accumulate extracellular Aβ deposits (Tajiri et al., 2013), but not Aβ plaques due to focal injury (Collins et al., 2015). Critically, age at injury also appears to affect the extent of these deposits (Nakagawa et al., 2000; Cheng et al., 2018). Despite increased APP expression, only increased Aβ (Hoshino et al., 1998) but not plaques have been reported in rodent models most likely due to the differences in rodent and human Aβ species. Diffuse Aβ plaques, however, were observed in a pig model with gyrencephalic brains similar to humans rather than lissencephalic brains as present in rodents upon DAI (Smith et al., 1999a).

As it relates to tauopathies, while repetitive injury models have been utilized to understand tau pathologies after injury, the effect of a single injury is yet to be elucidated. Presence of tau oligomers as well as tau phosphorylation has been reported in rat FPI (Hoshino et al., 1998; Hawkins et al., 2013), CCI (Acosta et al., 2017) and mouse blast injury (Huber et al., 2013) models as well as in tau transgenic mouse models (Tran et al., 2011b). Similar to studies that suggest an Aβ independent tau pathology in AD (reviewed here (van der Kant et al., 2020), tau phosphorylation due to injury in 3xTg mice has also been suggested to be independent of Aβ pathology (Tran et al., 2011a).

Despite the utility of animal studies, the inherent complexities and multi-cellular nature of the *in vivo* environment have made it difficult to make definitive mechanistic links between TBI-induced cellular injury and AD-related phenotypes as well as tease apart cell-autonomous vs. cell-non autonomous aspects of such relationships. Similarly, because animal models do not recapitulate all aspects of the human disease (Duff and Suleman, 2004; Seok et al., 2013; Warren et al., 2015), it is questionable to what degree these findings in animal models will translate to the human condition. In the future, the use of complimentary human-based systems will be necessary to confirm findings made using animal models.

## Potential Mechanisms for Traumatic Brain Injury-Induced Alzheimer’s Disease

**FIGURE 1:**
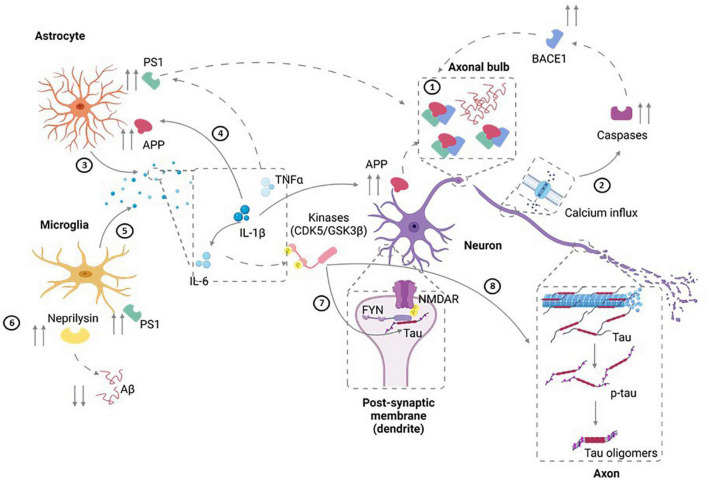
Potential Mechanisms for TBI-Induced AD. ➀ Diffuse axonal injury that results from TBI can lead to upregulation of APP as well as increased amyloidogenic processing of APP to pathogenic Aβ40 and Aβ42. In addition, axonal degeneration could also lead to the extracellular release of Aβ. ➁ Injury can also result in calcium influx in neurons. In turn, this can result in activation of caspases that induce the amyloidogenic processing of APP through elevated BACE1 availability. ➂ Injury-induced cytokine release (IL-1) by astrocytes can also result in ➃ the upregulation of APP expression and an associated increase in its amyloidogenic progressing through upregulation of BACE and PS1. ➄ Along similar lines, microglial activation and production of IL-6, IL-1 and TNFα can increase APP transcription, upregulation of PS1, and increased amyloidogenic APP processing. ➅ TBI also can modulate Aβ clearance by neprilysin generated by microglia leading to aberrant clearance of Aβ. ➆ Cell injury can also elevate FYN tyrosine kinase activity leading to increased tau phosphorylation as well as activation of the NMDAR subunit NR2B, thereby modulating synaptic plasticity and increasing excitotoxic vulnerability. ➇ IL-6 upregulation post-injury can also lead to tau hyperphosphorylation through elevated MAPK-p38 signaling. Figure was generated with the assistance of Biorender.

Despite the limitations of epidemiological, clinical, and animal-based studies, these investigations have suggested some potential mechanisms by which TBI can lead to AD onset and age-related progression. In this section, we will summarize some of these hypothesized mechanisms (Figure 1).

### Amyloid Beta(Aβ)

The amyloid cascade hypothesis posits that Aβ deposition is central to the pathogenesis of AD and might be due to either the increased production of amyloidogenic Aβ or due its aberrant clearance (reviewed here Selkoe and Hardy, 2016). Aβ peptides are generated from APP which is a transmembrane protein with an extracellular N-terminus and intracellular C-terminus. APP can be cleaved by the amyloidogenic pathway leading to Aβ peptide formation or by the non-amyloidogenic pathway. In the non-amyloidogenic pathway, α-secretase cleaves APP to form soluble APPα (sAPPα) and a membrane-bound C-terminal fragment called C83 (Kojro and Fahrenholz, 2005; LaFerla et al., 2007). In the amyloidogenic pathway, β-secretase cleaves APP to form soluble APPβ (sAPPβ) and a 99 amino acid C-terminal fragment called C99 which is further cleaved by y-secretase to form Aβ peptides of varying lengths and a membrane-bound APP intracellular domain (AICD). γ-secretase cleaves C83 formed by α-secretase cleavage to generate 3kDa protein called p3 (Haass et al., 1993) and a membrane-bound AICD. AICD formed by γ-secretase cleavage of C99 could be internalized and further cleaved by caspases to form toxic species (C31). Aβ peptides, predominantly Aβ42 are prone to aggregation (Kim and Hecht, 2005) and might form amyloid fibrils that accumulate to form senile plaques (Jarrett et al., 1993; Younkin, 1998). The exact species that is toxic to neurons is still unknown, although, several *in vitro* studies point toward Aβ oligomers being neurotoxic (Lambert et al., 1998; Hartley et al., 1999).

Diffuse axonal injury observed in TBI is marked by APP upregulation. Increased levels of Aβ40 as well as of Aβ42 and of Aβ deposits as a result of amyloidogenic processing of APP have been observed in animal models post injury (Hoshino et al., 1998; Smith et al., 1999a; Tran et al., 2011a; Tajiri et al., 2013). Various mechanisms of Aβ generation and accumulation have been posited-persistent axonal damage after a single injury might be a continual source of Aβ formation. Degeneration of these axons could release Aβ into the extracellular space. The accumulation of APP, BACE1 and PS-1 might also be due to the impaired axonal transport observed in DAI as suggested by a rodent peripheral nervous system (PNS) injury model (Cribbs et al., 1996; Chen et al., 2004). In fact, targeting these enzymes including BACE and γ-secretase has been shown to reduce neuronal cell loss post-injury (Loane et al., 2009).

APP expression could also be upregulated upon cytokine induction (IL-1) by neurons and glia observed post-injury (Goldgaber et al., 1989; Ciallella et al., 2002). Oxidative stress-induced upregulation of BACE and γ-secretase could also be speculated to contribute to the amyloidogenic processing of APP post-TBI (Tamagno et al., 2002). Activation of caspases due to calcium influx in neurons could also contribute to the amyloidogenic cleavage of APP potentially through increasing BACE availability (Walker et al., 2012). On the other hand, it is unclear if upregulation of APP post-injury has neuroprotective effects with cognitive outcomes declining upon knocking out APP (Corrigan et al., 2012a) or if it has no effect at all (Murai et al., 1998).

Excitotoxicity is a major secondary injury mechanism in TBI. N-methyl-D-aspartate receptor (NMDAR) activation post-injury has been shown to shift APP processing to the amyloidogenic pathway from non-amyloidogenic, potentially contributing to increased Aβ (Lesné et al., 2005). Upregulation of Aβ clearing enzyme neprilysin has also been observed in TBI patients who didn’t display Aβ plaque pathology indicating the possibility of Aβ clearance by neprilysin (Chen et al., 2009) and potentially an imbalance between the generation and clearance of Aβ in plaque formation. Recent studies indicate that promoting the non-amyloidogenic processing of accumulated APP by soluble APP alpha (sAPPα) administration (Corrigan et al., 2012b), increasing AB CA1 levels (Loane et al., 2011) and inhibiting c-JNK (Rehman et al., 2018) reduced Aβ pathology further suggesting a role for Aβ accumulation in AD pathogenesis post-TBI.

### Tau

Tau is a microtubule associated protein expressed in neurons whose primary function is to stabilize microtubules. It has six isoforms characterized by the number of tubulin binding domains (R) in the C-terminal and the presence of one or two or the absence of a 29-amino acid insert (N). Tau undergoes phosphorylation in at least 80 different sites, such as Thr231, Ser404 that allow it to detach from microtubules (Buée et al., 2000). Its phosphorylation depends upon the activity of various kinases such as MAPK (Drewes et al., 1992), CDK5 (Baumann et al., 1993) and GSK3β (Mandelkow et al., 1992) and phosphatases such as PP2A (Wang et al., 2013). Pathological hyperphosphorylation of tau leads to its oligomerization and formation of paired helical fragments that form the core of neurofibrillary tangles in AD. Furthermore, tau pathology has also been observed to spread across cells in a prion-like manner (Clavaguera et al., 2015).

Tau localizes mainly in axons, although low levels are present in dendrites as well (Ittner et al., 2010). Tau has been shown to associate with FYN tyrosine kinase in dendrites allowing FYN to phosphorylate and activate the NMDAR subunit NR2B, thereby regulating synaptic plasticity as well as potentially rendering neurons vulnerable to excitotoxicity (Salter and Kalia, 2004; Ittner and Götz, 2011). This has been speculated to contribute to Aβ induced toxicity in neurons in AD with FYN kinase inhibition suggested as a potential therapeutic for AD (Nygaard et al., 2014). FYN inhibition in a repetitive injury model has been shown to reduce tau phosphorylation and memory deficits (Tang et al., 2020) indicating a potential route for tau phosphorylation and pathology post-injury (Schumann et al., 2008; Rubenstein et al., 2017).

Tau pathology has been observed to spread to the contralateral hemisphere post-injury in a tau transgenic mouse model (Edwards et al., 2019). Prion-like transmission of tau pathology has also been observed in uninjured rodents upon injection of tau oligomers or brain homogenates from rodent models of injury (Gerson et al., 2016; Zanier et al., 2018). A reduction in phosphatases observed in rat injury models could also contribute to tau hyperphosphorylation (Arun et al., 2015). Additionally, tau phosphorylation post- injury has been suggested to alter the microglia and macrophage mediated inflammatory response hinting at a link between tau pathology and inflammation post-injury (Kokiko-Cochran et al., 2017).

### Inflammation

Chronic inflammation is a major component of AD pathology (Kinney et al., 2018). Recent GWAS studies in fact identify a number of immune responsive genes as risk factors for AD (Efthymiou and Goate, 2017). Inflammation has been shown to exacerbate Aβ and tau pathology as well as play a key role in neurodegeneration (Barroeta-Espar et al., 2019; Griciuc and Tanzi, 2021). While the inflammatory response to TBI is mediated by a number of cell types, we will focus on the microglial and astrocytic contribution.

Studies indicate that microglia phagocytose Aβ at the earlier stages of the disease contributing to its clearance but remain chronically activated marked by a decrease in their phagocytic capacity (Hickman et al., 2008; Yang et al., 2011). Such prolonged activation is linked to a chronic inflammatory response marked by impaired Aβ clearance, secretion of cytokines and reactive oxygen species (ROS) (Meda et al., 1995; McDonald et al., 1997). However, since markers for microglial activation have not been clearly established, there is some controversy on the nature of microglia surrounding Aβ plaques in AD post-mortem brains with reports of activated/reactive and of senescent microglia being present (Streit et al., 2009).

In brain trauma, microglia respond to injury by acutely elevating pro- and anti-inflammatory cytokine levels and by phagocytosing Aβ generated as a result of injury; in fact, microglia containing Aβ have been found in TBI patients (Chen et al., 2009; Mannix and Whalen, 2012). Acute release of pro-inflammatory cytokines have been shown to have neuroprotective effects (Penkowa et al., 2000). However, chronic activation of microglia has been observed in long-term survivors of TBI (Johnson et al., 2013) as well as in animal models of injury (Loane et al., 2014a) with the existence of primed microglia with exacerbated responses to immune challenges post-injury observed in a rodent model (Muccigrosso et al., 2016). Persistent microglial activation is hypothesized to contribute to neurodegeneration post-injury (Kokiko-Cochran et al., 2015). Upregulation of NADPH oxidase (NOX) enzymes that generate ROS post-injury (Loane et al., 2014a) could alter microglial activation by polarizing them to an M1-like state promoting neurodegeneration (Kumar et al., 2016).

Microglial activation and secretion of IL-6, IL-1, and TNFα by microglia and astrocytes have been suggested to increase APP transcription and y-secretase activity leading to amyloidogenic processing of APP (Sheng et al., 2003; Lee et al., 2008; Breunig et al., 2013). Additionally, upregulation of PS-1 in astrocytes and microglia has been observed in an animal model of injury (Nadler et al., 2008), suggesting glial role in Aβ processing. Moreover, Aβ accumulation post-injury has been shown to modulate microglial activation, with chronic activation observed in AD transgenic but not wild type (WT) mice after injury (Kokiko-Cochran et al., 2015). Upregulation of cytokines such as IL-6 (Kumar et al., 2015) reported after TBI could also lead to tau hyperphosphorylation, potentially through MAPK-p38 signaling (Quintanilla et al., 2004).

Additionally, metabotropic glutamate receptor activation in microglia has been suggested to decrease microglial activation as well as neuronal loss in a mouse model of injury (Byrnes et al., 2012; Loane et al., 2014b). However, the timing of inhibiting microglial activation might be important since a recent study suggests that inhibition of chronic microglial activation increases neurodegeneration post-injury in TBI patients (Scott et al., 2018).

## *In vitro* Models of Traumatic Brain Injury

Work with various model systems has allowed for the identification of several potential mechanisms by which TBI exerts its risk modifying effects as it relates to AD onset. As discussed previously each of these model systems has numerous limitations which make it difficult to dissect the mechanistic links between TBI and AD. As such, accessible *in vitro* models are needed to complement these existing model systems. In this section, we will review currently existing *in vitro* TBI models as it relates to mode of injury (e.g., transection, compression, stretch, blast, shear, microfluidic) and cell types (e.g., primary tissue, dissociated primary cells, immortalized cells lines, pluripotent cells) employed (Table 2). In addition, we will speculate how these models can be utilized in the future to study TBI-induced AD.

**TABLE 2:** Summary of *in vitro* injury model studies with key injury phenotypes observed in these models highlighted.

*In vitro* injury model	Cells/Tissue used	Key conclusions/Injury phenotype Observed	References
Compression	Primary rat neurons	Increased glutamate release and neuronal activity Transient changes in membrane permeability	Cullen et al., 2011; Tang-Schomer et al., 2014; Bar-Kochba et al., 2016
Shear	Primary rat neurons	Cell death and loss of neurites observed with high shear rates	LaPlaca et al., 2005
	Primary rat neuron and astrocyte co-culture	Astrocyte response to injury depends on injury severity-astrocytic hypertrophy and GFAP immunoreactivity observed	Cullen et al., 2007
Transection	Primary rat neurons	Acute increase in calcium influx post-injury	Mandolesi et al., 2004
		Altered dendrite numbers and length in interneurons	Brizuela et al., 2017
		Apoptotic cell death observed post-injury	Shah et al., 1997
Stretch	Primary rodent neurons	Increased calcium influx, disrupted axonal transport	Iwata et al., 2004; Lusardi et al., 2004; Li et al., 2019
	Cell line (NT2), primary rodent neurons	Axonal swelling observed, no primary axotomy but delayed elastic response observed after injury	Smith et al., 1999b; Tang-Schomer et al., 2010
	Primary rodent neurons	Transient increase in membrane permeability	Geddes et al., 2003
	Primary human neurons, hiPSC-derived neurons	Apoptotic and/or necrotic cell death observed	Sherman et al., 2016; Rosas-Hernandez et al., 2019
	Organotypic slice cultures, primary rodent neurons	Synaptic dysfunction and NMDAR activation observed	Tavalin et al., 1995; DeRidder et al., 2006; Cater et al., 2007

### Modes of Injury

Due to the heterogeneous nature of the type of injury that is induced in animal models, correlations between injury intensity and frequency with cellular phenotypes have been difficult to precisely ascertain (DeWitt et al., 2018). In this vein, various *in vitro* injury paradigms have been engineered which eliminate the complexities associated with *in vivo* experiments by minimizing confounding variables and facilitating a more direct investigation of the effects of mechanical insult. Here, we will discuss how each of these modes of injury recapitulate various aspects of a TBI. In addition, the limitations of each of these injury paradigms will be summarized.

#### Transection

Transection models involve utilizing stylets (Tecoma et al., 1989), blades (Chuckowree and Vickers, 2003) or rotating scribes (Mukhin et al., 1997) to introduce cuts in cultures, typically transecting axons. Although simple, the injury caused by these models are not often reproducible or controllable in terms of the biomechanical force involved. Moreover, they mimic primary axotomy in TBI, which occurs only in a small proportion of all TBI cases. Nonetheless, these models have lent insight into axonal regeneration post-injury (Chuckowree and Vickers, 2003), apoptotic cell death in mixed cultures (Shah et al., 1997) and calcium influx (Mandolesi et al., 2004) or proteomic changes (Lööv et al., 2013) following injury.

#### Compression

Compression injury models involve weight drop or pistons to compress 3-D, organoid or organotypic slice cultures (Tang-Schomer et al., 2014). Closed loop models allowing for reproducible injury have been developed (Cullen et al., 2011; Bar-Kochba et al., 2016). Additionally, these systems allow for live cell imaging. Importantly, controlling the piston size allows for the injury of various regions of the culture and study the effects in surrounding regions. However, the strain field is difficult to characterize with techniques to do so only being developed recently (Bar-Kochba et al., 2016).

#### Stretch

Stretch injury models involve culturing neurons on a flexible, deformable substrate (e.g., silicone) and stretching the substrate along one (uniaxial; Pfister et al., 2003) or both (biaxial; Morrison et al., 2006) directions using pneumatic systems. These models recapitulate DAI pathology and have shown axonal varicosities, axonal transport disruption and apoptotic cell death as observed in animal models or in clinical studies (DeRidder et al., 2006; Monnerie et al., 2010; Tang-Schomer et al., 2012). Additionally, these models allow for the culture of organotypic slice cultures, although typically 2-D cultures are utilized in these models. Moreover, these systems can be combined with multi-electrode array (MEA) systems to allow for the monitoring of electrophysiological changes post-injury (Kang et al., 2014). A disadvantage of these systems is that the applied strain is not uniform throughout the substrate and is not well characterized (Morrison et al., 2011).

#### Blast

Blast injury devices employ high pressure waves using compressed gas delivered via a shock-tube (Effgen et al., 2012) or using focused ultrasound (Lai et al., 2020) to mimic shock waves, particularly a transient increase in pressure due to a blast in 3-D cultures or organotypic slice cultures (Effgen et al., 2014; Zander et al., 2017). These have been used to study blood brain barrier disruption (Hue et al., 2013) and electrophysiological changes post-injury (Vogel et al., 2015). One limitation of these systems, though, is that the construction of these devices might be cost-prohibitive.

#### Shear

Shear stress models introduce shear stress either by moving one portion of the culture rapidly while its parallel surface is held fixed (LaPlaca et al., 2005) or by utilizing fluid shear stress by placing a rotating plate over cultures (LaPlaca and Thibault, 1997). Use of 3-D cultures in these models allows for complex-co-culture systems (Cullen et al., 2007). Critically, deformation due to shear stress is a major component of TBI and, thus, these systems uniquely mimic that aspect of the *in vivo* injury.

#### Microfluidic Platforms

More recently, microfluidic platforms to injure cells are also being employed to study axonal transport (Magdesian et al., 2012), hyperexcitability (Nagendran and Taylor, 2019), and mitochondrial damage (Dollé et al., 2014) post-injury. These devices typically culture cells in a microfluidic device patterned such that the neuronal cell bodies are restricted to one compartment and the axons are directed along a channel; some models also allow for culturing organotypic slice cultures on flexible substrates. Axotomy or injury is introduced by vacuum aspiration (Taylor et al., 2005), stretching the flexible substrate (Dollé et al., 2013) or compressing cultures using polydimethylsiloxane (PDMS) pads (Hosmane et al., 2011). Microfluidic platforms allow us to restrict the injury to specific regions of the cell (Shrirao et al., 2018). However, these systems require complex fabrication techniques as the pneumatic systems that often drive the compression/stretch require precise connections with the microfluidic chambers.

### Cells Utilized in *in vitro* Injury Models

Various cell and tissue types exist for the *in vitro* modeling of TBI. These cell types offer several advantages over their *in vivo* counterparts. For example, these cell types allow for a higher degree of control as specific cell types can be interrogated individually or combined at reproducibly defined ratios. In this vein, analysis of such cultures allows for the dissection of TBI-induced AD related phenotypes that are exerted through cell-autonomous vs. –non-autonomous mechanisms. In addition, injury with *in vitro* cell preparations allows for a greater ease-of-use than *in vivo* models which require technically complex surgical procedures. Finally, these *in vitro* cell types are amenable to a greater level of genetic and pharmacological manipulation than *in vivo* models. Despite these advantages, each of these cell preparations have inherent limitations that we will highlight in this section.

#### Primary Tissue Preparations

Acute preparations of rodent tissue (∼400 μm or less in thickness) can be isolated and subjected to injury within a few hours of isolation. These slices could be isolated from animals irrespective of their age, thereby allowing us to utilize mature cells in *in vitro* models. Additionally, these preparations preserve the native architecture of the tissue. However, the isolation procedure in itself could confound the injury response (Morrison et al., 2011).

On the other hand, organotypic slice cultures, isolated typically from rodent hippocampus are cultured for days before being used in an injury platform (often used in blast, stretch and compression models). Similar to acute preparations, these cultures retain the 3-D architecture and complexity of *in vivo* tissues. However, these slice cultures are generally isolated from younger animals, slowing down the maturation of the cells when cultured.

#### Dissociated Primary Cells

Primary cells enzymatically isolated from rodent tissue are used in several *in vitro* models (Shah et al., 1997; LaPlaca et al., 2005; Cullen et al., 2011; Bar-Kochba et al., 2016). These allow us to study cell-type specific responses to TBI as well as allow for complex co-culture models. Depending on the method, the isolation procedure introduces some mechanical damage prior to the *in vitro* injury. This is specifically an issue in case of cultures that utilize microglia that are transcriptionally different when isolated and cultured. Additionally, these cells are typically isolated from embryonic tissue and require long periods before maturation (Morrison et al., 2011). In addition, primary cell sources rapidly lose disease phenotypes upon *ex vivo* culture and are not amenable to genetic modification.

#### Immortalized Cell Lines

Various immortalized cell lines of various subtypes (e.g., neuronal-like, microglial, endothelial) and origins (e.g., human, mouse, rat) have been used extensively in conjunction with *in vitro* injury models (Smith et al., 1999b; Triyoso and Good, 1999; Pfister et al., 2003; Salvador et al., 2018; Li et al., 2019; Yin et al., 2020). The main advantage of these cell lines is their accessibility and extensive characterization. However, one major limitation of immortalized cells is that they might not represent the phenotypic maturity of their *in vivo* counterparts or display the same functional properties (Gordon et al., 2013; Carter and Shieh, 2015). In addition, immortalized cell lines often have abnormal karyotypes with unknown dosage at key disease-relevant genes (Ouellette et al., 2000).

#### Human Induced Pluripotent Stem Cells

Advances in cellular reprogramming have enabled the generation of *in vitro* central nervous system (CNS) disease-in-a-dish models that can be used to investigate the molecular mechanisms of disease origins as well as interrogate potential therapeutic interventions (Goldstein et al., 2015; Ghaffari et al., 2018). In particular, human induced pluripotent stem cells (hiPSCs), which can be derived from the reprogramming of somatic cells, can differentiate into all of the neural lineages and supporting cell types that comprise the CNS (Hong and Do, 2019). As such, hiPSC-derived cell types have been recently used in stretch and blast models of injury (Sherman et al., 2016; Lai et al., 2020), providing the ability to study TBI in a more relevant human system. However, hiPSC-derived cells resemble fetal neurons in nature, require prolonged differentiations to generate certain cell types and exhibit a lot of variability across clones and differentiations (Dolmetsch and Geschwind, 2011).

#### Use of *in vitro* Models to Study the Potential Mechanisms Linking Traumatic Brain Injury and Alzheimer’s Disease

Although *in vitro* models have been used extensively to interrogate the effects of TBI on neural cell phenotypes, only recently have these systems been applied to investigate the molecular and cellular mechanisms that might induce the onset of AD post-TBI. For example, Wu et al. (2020) used mouse hippocampal slice cultures in conjunction with a weight drop model to interrogate the TBI-induced AD-related pathologies. Interestingly, the authors observed that injury induced a marked increase in APP cleavage. Mechanistically, the authors determined that injury induces increased delta-secretase (AEP) expression which mediates APP fragmentation and subsequent neuronal cell death.

Several studies have also used *in vitro* platforms to investigate the potential mechanisms by which cell injury can lead to tau-related pathologies. For example, in one such study a stretch model employing rodent hippocampal cells was used to investigate the effect of injury on tauopathy (Braun et al., 2020). This study revealed that mechanical stretching of cultured neurons resulted in tau mislocalization to dendritic spines which results in subsequent synaptic dysfunction. Critically, the authors identified a strong relationship between injury dynamics and the extent of tau mislocalization. Finally, through the use of pharmacological inhibitors the authors showed that the injury-induced synaptic deficits due to tau hyperphosphorylation were mediated likely by GSK3β and CDK5, kinases that phosphorylate tau and whose expression has been observed to be upregulated in AD brains (Yamaguchi et al., 1996; Blalock et al., 2004).

Collectively, these studies set a strong precedent for using *in vitro* models for the identification of possible mechanisms linking TBI and AD. In the future, given the utility of neurons, astrocytes, and microglia differentiated from patient derived hiPSCs in various culture formats, these cells could be utilized in *in vitro* injury models to investigate the mechanisms of TBI-induced AD. In one such study, a high-intensity focused ultrasound was used to induce mechanical injury in 3-D hiPSC-derived cortical organoids. Remarkably, injured organoids displayed increased levels of pathologically associated phosphorylated tau (Lai et al., 2020). In addition, injury disrupted nucleocytoplasmic transport in a manner similar to that observed in AD (Eftekharzadeh et al., 2018). Finally, a more recent study that used hiPSC-derived neurons in the context of a stretch-based model demonstrated that injury reduced APP axonal transport as well increased the accumulation of axonal amyloidogenic fragments (Chaves et al., 2021). Moving forward, given that phenotypes such as elevated Aβ peptides and tau hyperphosphorylation are readily observed in AD hiPSC lines (Israel et al., 2012; Kim et al., 2015; Jones et al., 2017), future studies could employ these cell lines to investigate the effect of cell injury on the induction or augmentation of AD-related molecular, biochemical, and cellular changes.

## Future Directions: Role of Sex and Genetic Factors

There is emerging evidence that sex can have a significant influence on TBI risk and related clinical outcomes (Gupte et al., 2019; Ma et al., 2019). The potential genetic, biochemical, and environmental causes for these sex-specific differences have been reviewed elsewhere (Gupte et al., 2019; Ma et al., 2019). On the other hand, there is a paucity of research related to investigating the mechanisms by which TBI-induced AD can be influenced by sex. Interestingly, while AD is more prevalent among females, the clinical outcomes associated with TBI appear to be worse in males (Viña and Lloret, 2010; Laws et al., 2018; Gupte et al., 2019; Ma et al., 2019; Guo et al., 2022). Thus, future studies that employ *in vivo* and *in vitro* models might be able to identify the molecular underpinnings of sex-based differences. Indeed, several studies have used hiPSC-based models of other diseases to identify the role of sex in disease onset and progression (Huo et al., 2019; Lock et al., 2021; Paci et al., 2021). In the same regard, moving forward similar study designs could be employed to determine the presence and mechanisms of sex-based differences in TBI-induced AD.

In addition to age at injury and injury severity, the pathological consequence of TBI can be influenced by a variety of genetic factors (Wilson and Montgomery, 2007; Dardiotis et al., 2010; Bennett et al., 2016; Kurowski et al., 2019). As it relates to TBI-induced AD, polymorphisms in Apolipoprotein E (APOE) appear to be the most prominent risk-modifying genetic factor. APOE is a cholesterol transport lipoprotein that is primarily secreted by astroglial cells in the CNS. Broadly speaking, APOE has three main isoforms (E2, E3, and E4) of which APOE4 has been reported to increase the risk as well as decrease the median age of AD onset whereas APOE2 has been demonstrated to mitigate the onset and age-related progression of AD (Bales et al., 1997; Castellano et al., 2011). The role of APOE in modulating AD-related phenotypes has been reviewed extensively elsewhere (Liu et al., 2013). Briefly, APOE isoforms have been shown to differentially clear Aβ and affect its aggregation (Bales et al., 1997; Castellano et al., 2011) as well as modulate tau phosphorylation and immune responses in AD (LaDu et al., 2001; Brecht et al., 2004).

Despite the strong evidence linking APOE isoforms to modulation of AD onset and age-related progression, the role of APOE polymorphism in influencing AD-related outcomes post-TBI remains unclear. For example, several studies have indicated that clinical outcomes worsen in APOE4 individuals post-injury with Aβ deposition being observed more frequently (Nicoll et al., 1995; Friedman et al., 1999). Confirmatory work with a transgenic AD mouse model demonstrated that in ApoE4 animals injury induced Aβ plaque formation whereas only diffuse Aβ deposits were observed in ApoE3 or ApoE knockout mice indicating a role for ApoE4 in Aβ aggregation (Hartman et al., 2002). On the other hand, in a 3xTg AD mouse model crossed with human ApoE (ApoE2, 3 and 4 isoforms), increased APP immunoreactivity, but similar levels Aβ40 or Aβ42, were observed acutely after injury in 3xTg-ApoE4 mice compared to mice with other ApoE genotypes (Bennett et al., 2013). However, injury outcomes have been reported to worsen with time in human ApoE4 expressing mice so evaluation of Aβ and tau pathology at further time points are required (Sabo et al., 2000). Interestingly, a recent transcriptomic study addressing this question observed no ApoE isoform specific changes in the transcriptome post-injury in human ApoE expressing mice (Castranio et al., 2017). However, the isoform specific effects may also be brain-region dependent (Ezra et al., 2003) with gene expression changes observed in an isoform-dependent manner to a larger extent in the hippocampus (Crawford et al., 2009). Some epidemiological studies have found no additional risk in developing AD conferred by ApoE4 after TBI with one study indicating age-dependent effects of ApoE in determining injury outcome (Teasdale et al., 2005) suggesting the need for further studies elucidating the role of ApoE in determining injury outcome as well as in mediating neurodegenerative processes post-injury (O’Meara et al., 1997; Chamelian et al., 2004).

As it relates to the use of *in vitro* models, recent studies have demonstrated the utility of hiPSC-based platforms combined with powerful gene editing techniques such as CRISPR/Cas9 in investigating the contribution of genetic risk factors to disease onset and progression. With respect to APOE, isogenic hiPSCs to investigate the mechanisms by which APOE4 increases and APOE2 decreases AD risk (Lin et al., 2018; Wang et al., 2018; Brookhouser et al., 2021; Martens et al., 2021; Sienski et al., 2021) can be used in conjunction with *in vitro* injury models to determine the combinatorial effect of APOE isoforms and cell injury on the manifestation of AD-related phenotypes. In addition, numerous genome-wide association studies (GWAS) studies have identified several risk factors associated with altered probability of AD onset (Bettens et al., 2010). In fact, some of these genes, such as *BDNF*, *IL-1*, and *p53*, have also been identified to influence clinical outcomes post-TBI (Dardiotis et al., 2010; Cortes and Pera, 2021; Zeiler et al., 2021). In addition, many of the genetic risk factors that affect clinical outcomes post-TBI play critical roles in pathways (e.g., inflammation, microglia activation, neurotransmitter synthesis, synaptic formation) that are dysregulated in AD (Dardiotis et al., 2010; Cortes and Pera, 2021; Zeiler et al., 2021). Moving forward, hiPSC-based isogenic models employed with *in vitro* injury systems can be used to investigate the influence of these additional genetic risk factors on the development of AD-related pathologies post-injury.

## Conclusion

While there is some conflicting evidence for TBI-induced AD in epidemiological studies, clinical studies and animal models suggest a strong link between the two. Pathologies observed in AD including Aβ deposition, hyperphosphorylated tau and persistent inflammation have been observed in a fraction of TBI patients as well as in animal models. The mechanisms underlying the development of such pathologies are yet to be elucidated—injury induced amyloidogenic processing of APP, dysregulation of kinases phosphorylating tau and chronic inflammation mediated by microglia are some of the major avenues currently being explored. While transgenic rodent models have lent valuable insight into these mechanisms, several *in vitro* models developed to mimic aspects of traumatic injury could be leveraged to further probe the link between TBI and AD. Nonetheless, as the famous statistician George Box stated “All models are wrong, but some are useful.” Thus, moving forward researchers could utilize the complementary strengths of *in vivo* and *in vitro* systems to address the underlying causes of TBI-induced AD and identify potentially novel therapeutic targets.

## Author Contributions

GS and DB collected, analyzed, and interpreted current literature, and wrote the manuscript. Both authors contributed to the article and approved the submitted version.

## Conflict of Interest

The authors declare that the research was conducted in the absence of any commercial or financial relationships that could be construed as a potential conflict of interest.

## Publisher’s Note

All claims expressed in this article are solely those of the authors and do not necessarily represent those of their affiliated organizations, or those of the publisher, the editors and the reviewers. Any product that may be evaluated in this article, or claim that may be made by its manufacturer, is not guaranteed or endorsed by the publisher.
